# A Latent Class Analysis of Reproductive Coercion Experiences Based on Victim-Survivors’ Acknowledgment and Disclosure Patterns

**DOI:** 10.1177/08862605241259409

**Published:** 2024-06-19

**Authors:** Sylvie Lévesque, Arianne Jean-Thorn, Catherine Rousseau

**Affiliations:** 1 Université du Québec à Montréal, Canada; 2 Université d’Ottawa, ON, Canada

**Keywords:** reproductive coercion, acknowledgment, reproductive autonomy, disclosure, gender-based violence

## Abstract

Reproductive coercion (RC) is a form of violence involving behavior that interferes with an individual’s contraceptive and reproductive decisions. Like other forms of violence perpetrated by intimate partners, victims of RC do not necessarily identify it as such. Similarly, victim-survivors do not readily disclose their experiences or seek support and treatment. This study identifies patterns of acknowledgment and formal and informal disclosure of RC experiences in a community sample of 317 participants. Latent classes are then compared with respect to characteristics of victims/survivors, RC consequences, and associated contexts. Participants completed measures to assess experiences of RC and violence perpetuated by intimate partners as well as social support, posttraumatic stress symptoms, and consequences for psychological and sexual health. Latent class analysis was performed to identify acknowledgment and disclosure patterns. An optimal three-class solution was selected: High unacknowledgment with ambivalence, High disclosure (41%); High acknowledgment, High disclosure (30%); and Hesitant acknowledgment, No disclosure (29%). Classes were identified according to the presence of social support, living with a disability, victimization experiences, and mental and sexual health consequences. Future studies should explore the relationship between RC acknowledgment and disclosure, which can influence victims’ search trajectories for support and services.

## Background

Reproductive coercion (RC) refers to violent behavior against women that specifically targets reproductive autonomy (Grace & Anderson, 2018). Such behavior interferes with women’s autonomous decision-making in matters of birth control and family planning (Miller et al., 2010; Moore et al., 2010). Behaviors may be enacted by an intimate partner (Grace & Anderson, 2018), but also by family members and in-laws (Gupta et al., 2012; Silverman & Raj, 2014), and even medical professionals (Stote, 2012).

Miller et al. (2010) proposed a reproductive coercion scale (RCS) with two categories of behavior: birth control sabotage (e.g., damaging condoms, hiding or throwing out birth control pills) and pregnancy coercion (forcing pregnancy continuation or termination). Recent studies have also considered abortion coercion (forcing or impeding access to an abortion) and the use of pressure or threats to force pregnancy continuation, collectively called control of pregnancy outcomes, which is considered the be the third category (Moulton et al., 2021; Nikolajski et al., 2015). Although most of the research on RC divides the manifestations into three categories, a more recent conceptualization proposal by Sheeran et al (2022) offers an understanding of RC and abuse as being oriented either toward the promotion of pregnancy or its avoidance (pregnancy prevention). The manifestations would therefore be oriented toward (a) forced or pressurized conception; sabotage of contraception; (b) forced or pressurized continuation of pregnancy; (c) forced or pressurized contraception; sterilization; and (d) forced or pressurized abortion.

Other recent reviews have focused on behaviors associated with contraceptive resistance, examining their similarities and differences. Gómez-Durán and Martin-Fumado (2024) conducted a rapid review of stealthing, which they refer to as nonconsensual condom use deception. Their review identifies the main perceived or reported motivations for stealthing: sexual pleasure, domination over the partner, intentional STI transmission, and RC. They argue that nonconsensual condom use deception should not be categorized under CR and that motivations should not be part of its definition.

Chen et al. (2024) present a scoping review on condom use resistance (CUR), highlighting its commonalities and differences with RC. They explain that coercive CUR involves resisting condom use without specifying motivations, whereas CR involves controlling reproductive outcomes through various behaviors. Chen et al. (2024) also note that certain behaviors can overlap between CUR and CR and emphasize the challenge of studying behaviors from the perspective of the affected person, who may be unaware of the motivations behind these actions.

The available Canadian data reveal that, in a community sample of 427 women and nonbinary individuals, 62.8% had experienced contraceptive sabotage (i.e., failure to withdraw before ejaculation, forcing unprotected sex, nonconsensual condom removal) in their lifetime, while 14.1% had experienced at least one pregnancy coercion behavior and 9.8% had had a partner who attempted to control a pregnancy outcome (Lévesque, Rousseau, Jean-Thorn, et al., 2023). However, not all individuals who experience RC define the acts as such. Although RC is a newly documented form of violence in the research and has only recently gained legal recognition, at least in Canada (R. v. Hutchinson, 2014; R. v. Kirkpatrick, 2022), the definitions that enable individuals to acknowledge that violence has occurred remain unclear (Brodsky, 2017; Nikolajski et al., 2015). Some individuals can recognize behaviors as violent and attribute the responsibility to the aggressor, but others blame themselves for what is done to them, and still others identify the situation as the result of poor communication with their intimate partner (Harned, 2005; Johnstone, 2016).

### Acknowledgment of Violence Based on Gender and RC

Many victims of violence by an intimate partner have difficultsuy calling the behavior violent. Thus, based on the testimonies of women living in a shelter for victims of domestic violence or who used the shelter’s services, one study noted that acknowledgment of the violent nature of an intimate partner’s behaviors was like a “wavering line in the sand” that is easily erased by the tide (Coggins & Bullock, 2003). The women said they could identify behaviors as being violent and intolerable, but tended to push the boundaries of tolerance when they occurred in an intimate context (Coggins & Bullock, 2003). In other words, their line in the sand could disappear, and their stance on what was intolerable in an intimate relationship could shift. These researchers also observed that some female victims, if certain behaviors they identified as intolerable were not enacted, found it easier not to qualify other behaviors that their partner enacted as violent.

Ambivalence about what is and is not violent behavior has been reported most often in studies of sexual violence and assault (Fisher et al., 2003; Harned, 2005; Koss, 1985). Harned’s (2005) qualitative study revealed that some women were uncertain about how to label their experiences. These women were unsure whether they had consented to the sexual acts performed, what type of violence they had experienced, and the impact on their well-being. In this sense, the acknowledgment of violent behavior would be a gradual process over time as suggested in the study of (Harned, 2005). This process could be influenced by a variety of factors, including widespread social movements denouncing violence against women (e.g., #MeToo, #BeenRapedNeverReported). Nevertheless, violence remains difficult to acknowledge when it occurs in an intimate relationship (Clements et al., 2021). RC behaviors are particularly hard to acknowledge when the woman has an emotional bond with the perpetrator, or when the violence is not physical in nature (Lévesque & Rousseau, 2019; Moore et al., 2010). The controlling intimate partner might also persuade a woman to doubt her understanding of the behavior (gaslighting), or the victim might define the acts as not being violent to avoid blaming herself, her children, or her intimate partner (Moore et al., 2010).

Lipinski et al. (2021), in a study of sexual violence experienced by 131 college women, proposed three classifications of rape survivors: a group who were ambivalent about acknowledging what happened (28.2% of the sample), a group in which rape was acknowledged (49.6%), and a group in which rape was unacknowledged (22.1%). Ambivalence was associated with significantly higher posttraumatic stress symptoms compared to the unacknowledged group (Lipinski et al., 2021). Thus, acknowledgment of the violent nature of the behavior appeared to influence the well-being and mental health of the victims.

The available data on acknowledgment is based mainly on sexual violence situations, where the perpetuator is not necessarily an intimate partner in a committed relationship. However, it is important to consider the issue of RC acknowledgment in order to understand subsequent support- and service-seeking trajectories.

### Formal and Informal Support for Victim-Survivors and the Consequences of RC

Studies have demonstrated the importance of informal support to help victims make sense of what they have undergone. Sharing their experiences enables them to initiate the acknowledgment process, which is associated with higher psychological well-being (Clements & Olge, 2009). Informal support and validation of the victimization experience provided by others can also encourage women to acknowledge violence (Bujold & Lévesque, 2022; Lévesque & Rousseau, 2019). This feedback may help women feel believed and accepted, and that their feelings are acknowledged. Furthermore, hearing others name what they experienced as violence can influence their perceptions of what happened (Lévesque & Rousseau, 2019). On the other hand, negative reactions by others can encourage victims to doubt the violent nature of the behavior or to blame themselves for it (Bujold & Lévesque, 2022). This informal support is critical when it is unsafe for victims to seek healthcare (Moulton et al., 2021). For example, some women victims of RC ask a female friend to help them access sexual and reproductive health services (e.g., drive them there and make a secret appointment) or to obtain less detectable contraceptive means (e.g., Intrauterine devices [IUD], Depo-Provera, morning-after pill) (Boyce et al., 2020; Grace et al., 2020). However, studies in this area provide little information on RC disclosure and requests for support from family and friends.

RC events have many consequences for mental, physical, and sexual health, including unwanted pregnancy; STIs mental health symptoms associated with depression, stress, and anxiety; feelings of guilt and shame; hypervigilance and recurring nightmares; and reduced desire and sexual pleasure (Grace & Miller, 2023; Moulton et al., 2021; Nikolajski et al., 2015). Victims who do not acknowledge behavior as violent may present at support services with symptoms that appear unconnected to violence (e.g., chronic pain or somatization, depression, anxiety disorders, posttraumatic stress disorder, eating disorders, sleep disorders, alcohol, and other substance use disorders, suicidal and self-harm behavior) (Stewart et al., 2021). When women go to a health care or social services center, they may not disclose that they were subjected to RC (Lévesque, Rousseau, Raynault-Rioux, et al., 2023). They generally want immediate treatment for an urgent physical health problem (e.g., STI test, morning-after pills, pregnancy tests, and abortion) (Kazmerski et al., 2015). Nevertheless, studies show that women may disclose what happened during the consultation if the attending staff ask the right questions (Chamberlain & Levenson, 2012; Lévesque & Rousseau, 2021). Unfortunately, few healthcare professionals ask the questions that would uncover RC (Tarzia et al., 2019).

### The Present Study

This study aimed to better understand the complex associations between patterns of RC acknowledgment based on Lipinski et al.’s (2021) taxonomy (acknowledgment, ambivalence, and unacknowledgment) and disclosure of RC behavior to family, friends, and healthcare professionals. Latent class analysis (LCA) is relevant for this purpose because it allows the creating, within a heterogenous population, of subgroups that share similar characteristics based on pre-identified indicators. Thus, this study attempts to distinguish, in a general population, diverse patterns of RC acknowledgment and formal and informal disclosure and to identify the consequences associated with these patterns. We also wanted to explore how sociodemographic variables, social support, lifetime prevalence of intimate partner violence (IPV), and different forms of RC experiences were associated with acknowledgment and disclosure patterns. This study contributes to the knowledge by responding to the four following questions: (a) Are there distinct groups of victim-survivors based on patterns of RC acknowledgment and disclosure to family and friends or health care professionals?; (b) Are there significant differences among these subgroups in terms of demographic and psychosocial factors?; (c) Are there subgroup differences in terms of victims-survivors’ victimization experiences?; and (d) Are there subgroup differences in terms of mental and sexual health?

## Methods

### Procedure and Participants

All study procedures obtained ethical approval by the research ethics committee of the first author’s (S.L.) university. Participants were recruited mainly via a call for participation on social networks. The call was extended to the partner organizations (*Fédération québécoise pour le planning des naissances* and Planned Parenthood Ottawa) and to community and advocacy organizations. To be included in the study, participants had to be between age 18 and 55, have been assigned female status at birth, and have been in an intimate relationship with someone who could have made them pregnant.

Participants were asked to read and agree to a consent form. Refusal to participate led directly to questionnaire end. Those who agreed to participate completed an online questionnaire hosted on a secure Qualtrics platform. The questionnaire was available in French and English from September 2020 to April 2021. A list of support resources was provided at questionnaire’s start and end to enable access if needed. Participants who completed the entire questionnaire could opt to enter a contest to win one of five prizes worth $50. Winners were randomly selected.

A total of 493 responses were recorded on the platform. Once ineligible individuals, incomplete responses, duplicates, and non-serious responses (e.g., response times too short) were removed, 427 responses remained in the database. To be included in analysis, individuals had to have experienced at least one lifetime episode of RC. The final sample comprised 317 participants.

### Measures

#### Reproductive Coercion

We built on previous scales (Katz et al., 2017; Miller et al., 2010, 2014) to develop an RC measure that incorporates new behaviors documented in recent qualitative studies (Grace et al., 2020; Moulton et al., 2021). The measure includes the RC items proposed by Miller et al. (2010) translated into French and then back-translated into English (Corbière & Fraccaroli, 2014). The two English versions were then compared for consistency and minor changes were made. The new measure also includes items that specifically address control and monitoring of contraceptive use, imposition of long-term or permanent contraception, imposition of abortion against the pregnant person’s will, and pressure to give a child up for adoption after delivery. The items were also modified to include trans and nonbinary individuals as well as sexual orientations. The first version of the measurement tool was validated by the family planning community partners involved to ensure item clarity. The questionnaire was adjusted in light of their feedback. It was then pre-tested by four volunteers with different backgrounds and life experiences, and their comments were integrated before the questionnaire was launched.

All items were formulated in terms of lifetime prevalence. Participants responded to seven items on contraceptive sabotage (e.g., “Has an intimate partner ever . . . prevented you from using a birth control method (e.g., condom, birth control pills) or prevented you from going to the clinic to get birth control?”; “ . . . removed a condom without telling you while you were having sex?”). Three items addressed pregnancy coercion (e.g., “. . . threatened to leave you or damage your reputation if you become pregnant?”). Eight items addressed control of pregnancy outcomes (e.g., “. . . threatened you so that you would get an abortion?”; “. . . threatened you so that you would continue a pregnancy?”). Responses were dichotomous (yes/no). Internal consistency showed adequate results (*α* = .75, *ω* = .76).

#### Acknowledgment Items

To measure acknowledgment of RC events, an inventory was compiled to represent different interpretations of the experiences. These items were initially drawn from qualitative studies on individual understandings and descriptions of such events (LeMaire et al., 2016; Lévesque & Rousseau, 2019). Further items were added based on existing acknowledgment scales used in sexual violence studies (Littleton et al., 2017; Wilson & Miller, 2016). The resulting nine items were then reviewed for relevance in an RC context. Participants responded to the items only if they reported having experienced at least one RC event. Four items assessed their tendency to acknowledge experiences as violent. Participants were asked to consider all situations and behaviors that involved an intimate partner, including: “lack of consideration for my choices/my person in the sexual relationship,” “rape or sexual assault,” “domestic violence or control,” and “some type of crime, but not sure what type.” Four other items assessed the tendency not to acknowledge RC: “Were they . . . miscommunications or misunderstandings?”; “. . . normal situation(s) in a couple or in an intimate relationship?”; “. . . about your partner’s desire to have a child?”; and “. . . about your partner getting involved in birth control and family planning?” A ninth item was added to provide a general “ I don’t know” for all the items, expressed as: “I don’t know how to describe the event(s).” This enabled measuring ambivalent acknowledgment of the events as RC. Participants could check as many of the eight items as they wished. Thus, they could check both acknowledgment and unacknowledgment items and the ambivalence items.

All items were recoded into a three-point Likert scale: No (0), Yes (1), and Don’t know (998). Three dichotomous scores (0–1) were then derived from the data to determine the presence of acknowledgment, unacknowledgment, and ambivalence. Note that participants could receive scores for all three responses. Scoring and coding procedures are described in the Supplemental Material.

#### Disclosure

Participants responded to two questions about RC disclosure to family and friends or professionals: “Have you talked about these events with someone you feel close to and trust?”; and “Have you talked to a health professional, social services professional, or psychosocial worker about these events?.” The following instruction was added: “Note: the professional could be a nurse, doctor, psychologist, sexologist, or social worker. The consultation could be in person, over the telephone, by text message, or by video conference.” Participants responded No (0) or Yes (1).

#### Psychological and Sexual Health Consequences

With reference to the RC items, participants were also asked if they had suffered any consequences for their psychological, sexual, or reproductive health while considering the totality of the experiences rather than specific RC events. A 16-item inventory of consequences was adapted from the General victimization—Impact on respondent module (Statistics Canada, 2014). Ten items addressed psychological consequences (e.g., “You were angry”; “You had anxiety/anxiety attacks”; “You felt depressed”), with five items on sexual and reproductive health consequences (e.g., “You experienced a loss of sexual interest or sexual desire”; “You have been diagnosed with an STI”) and one item for no consequences (“You were not affected”). Participants could check any or all items that applied. Two dichotomous scores were derived: a psychological consequence score and a sexual and reproductive health consequence score. If one or more items were checked for either consequence type, participants were considered as having this consequence type (scored as 1). If participants did not check any consequences or indicated that they were not affected, they were considered as having no consequences (scored as 0).

#### Posttraumatic Stress Symptoms

Posttraumatic stress symptoms were assessed with the Primary Care Post Traumatic Stress Disorder (PTSD) Screen (Prins et al., 2003). It comprises four items, such as, “You had nightmares about the event(s) or you thought about it when you didn’t want to.” Participants responded with No (0) or Yes (1) to each item. A dichotomous score was created: a No response to two items or less was scored as 0 (*low level of PTSD symptoms*). A positive response to three items or more was scored as 1 (*high level of symptoms*). The measure showed acceptable reliability (*α* = .78, *ω* = .78).

#### Intimate Partner Violence

Five items adapted from the Composite Abuse Scale (Hegarty et al., 2005) and the Composite Abuse Scale (Revised)—Short Form (Ford-Gilboe et al., 2016) assessed lifetime prevalence of IPV. Participants responded Yes (1) or No (0) to each item (e.g., “In your lifetime, in an intimate relationship, have you ever been insulted, despised, or humiliated by your partner?”). Item scores were summed to calculate a continuous score. A dichotomous score was then created to indicate experience (scored 1) or not (scored 0) of lifetime IPV. Internal consistency showed adequate results (*α* = .75, *ω* = .76).

#### Social Support

Five items from a study on parental attitudes and family behaviors in Québec (Institut de la statistique du Québec, 2019) were used to assess the participants’ social support network. A sample item is, “If something went wrong, someone would help me.” Participants responded on a four-point Likert scale ranging from *Strongly Disagree* (1) to *Strongly Agree* (4). Two items were reverse coded such that higher scores indicated greater social support. Item responses were summed to calculate the total score. A dichotomous score was then created based on a clinical threshold at the 80th percentile, as set by the Institut de la statistique du Québec (2019) (in our sample, a score of 20). The scale showed excellent reliability (*α* = .91, *ω* = .91).

#### Sociodemographics

Participants completed a sociodemographic questionnaire on gender, age, sexual orientation, relational status, occupation, and educational background. They were also asked if they were indigenous (i.e., “Do you consider yourself part of a First Nations, Métis, or Inuit community?”), if they were a visible minority (i.e., “In Canada, are you perceived as a member of a minority group because of cultural or physical characteristics?”), and if they were living with a disability (i.e., “Do you consider yourself to be living with a visible or non-visible disability*?*”).

### Analysis Approach

A LCA was performed in MPlus 7.4 (Muthén & Muthén, 1998–2017). This method allows identifying subgroups within populations based on a set of behaviors or characteristics while using a robust standard error and accounting for non-normally distributed data (Lanza & Cooper, 2016). All Acknowledgment and Disclosure items were used as indicators. We tested different numbers of classes to identify the best class solution. Multiple fit indices were then compared to select the model that best fit the data. The Akaike information criterion (AIC; Akaike, 1987), the Bayesian information criterion (BIC; Schwarz, 1978), and the adjusted Bayesian information criterion (aBIC; Sclove, 1987) were used to compare class solutions. For all three indices, lower values indicate better fit. To ensure distinct patterns, entropy was also determined. Entropy closer to 1 indicates better fit. The Lo-Mendell-Rubin likelihood ratio (LMR LR) test and the Bootstrapped likelihood ratio test (BLRT) were also used to determine whether a solution provided a better fit than the *n* − 1 model and to ensure parsimony of the selected model (Lo et al., 2001). A significant *p*-value for both tests indicated that the hypothesized model with one extra class provided better fit than the model with one less class.

Once the final model was selected, multinomial logistic regressions were conducted to identify which variables (sociodemographic characteristics, PTSD symptoms, lifetime experience of RC and the psychological and sexual health consequences, lifetime IPV, and social support) were associated with latent class membership. The regression analysis was performed using the R3step approach in MPlus, which has been demonstrated preferable to other methods in the literature (Asparouhov & Muthén, 2014), and which allows controlling for latent class misclassification. A reference class was selected based on the pattern that generated optimal well-being, that is, an acknowledgment and disclosure pattern. We then compared the two remaining classes using the second-best reference class selected in the same way as the first.

## Results

The final sample comprised 317 participants who reported experiencing at least one lifetime RC event, given that only those who had experienced at least one responded to the acknowledgment and disclosure items. The sample contained mainly cisgender women (91.8%), who self-identified as heterosexual (56.2%) and were born in Canada (84.2%). Over one-third of participants had completed undergraduate studies (40.7%) and over two-thirds were in a relationship with a regular partner (67.8%). Most participants (83.3%) reported at least one experience of contraceptive sabotage, with 17.7% at least one pregnancy coercion and 12.6% at least one control of pregnancy outcomes. Table 1 presents the sociodemographic information.

**Table 1.** Sociodemographic Characteristics of the Sample (*n* = 317).

Variable	*N*	%
Age	*M* = 29.56	*SD* = 6.84
Gender^a,b^		
Woman	291	91.8
Man	2	0.6
Nonbinary	7	2.2
Genderfluid	5	1.6
Agender	4	1.3
Two-spirit	2	0.9
Questioning	5	1.6
Sexual orientation		
Heterosexual	178	56.2
Homosexual	7	2.2
Bisexual	66	20.8
Asexual	2	0.6
Pansexual	38	12
Questioning	26	8.2
Education^b^		
High school or college	103	32.5
Undergraduate and graduate university studies	195	61.5
Occupation^b^		
Student	104	32.8
Worker	184	58.0
Unemployed	9	2.8
Visible or non-visible disability^b^		
Yes	47	14.8
No	246	77.6
Member of an ethnocultural minority in Canada^b^		
Yes	21	6.6
No	273	86.1
First Nations, Métis or Inuit^b^		
Yes	6	1.9
No	292	92.1

a All participants were assigned female at birth.

b *n* may not sum up to 317 due to missing data.

### Research Question 1: LCA of Acknowledgment and Disclosure of RC

Table 2 presents the fit indices for the 2- to 5-class LCA models. The 2- and 3-class solutions provided the best model fit, with the lowest BIC and the highest entropy and with significant LMR LR and BLRT. The 3-class solution had the lowest aBIC, with significant LMR LR and BLRT. The 5-class solution presented the lowest AIC, but all other fit indices showed poor fit. The 3-class solution appeared to be the best, with most fit indices adequate. Moreover, a 3-class solution was considered optimal in the literature (Lipinski et al., 2021).

**Table 2.** Model Fit Indices for the Latent Profile Analysis of Participant’s Acknowledgment and Disclosure.

Model	AIC	BIC	aBIC	Entropy	LMR LR	BLRT	Class Proportions (%)
2-Class LCA	1,625.155	**1,666.503**	1,631.613	**0.98**	***p* < .001**	***p* < .001**	67-33
3-Class LCA	1,609.014	1,672.915	**1,618.995**	0.78	***p* < .05**	***p* < .05**	41-30-29
4-Class LCA	1,611.887	1,698.342	1,625.392	0.9	*p* = n.s.	*p* = n.s.	39-32-11-18
5-Class LCA	**1,605.599**	1,714.607	1,622.626	0.68	*p* = n.s.	*p* = n.s.	18-37-18-20-7

*Note.* AIC = Akaike information criterion; aBIC = adjusted Bayesian information criterion; BIC = Bayesian information criterion; BLRT = bootstrapped likelihood ratio test; LCA = latent class analysis; LMR LR = Lo-Mendell-Rubin likelihood ratio; n.s. = not significant. Bold values denote statistical significance at the *p* < 0.01 or *p* < 0.05 level.

**Figure 1.**
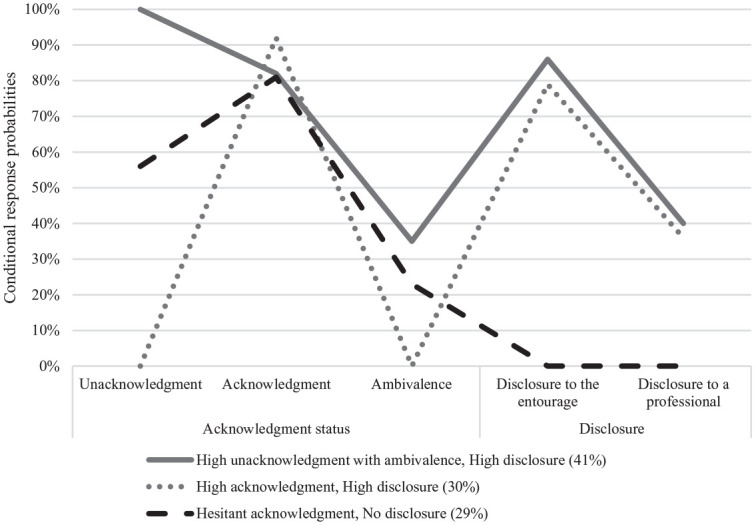
Patterns of acknowledgment disclosure (*n* = 317).

Figure 1 presents the 3-class solution based on the model’s estimated probabilities for endorsement of the acknowledgment and disclosure indicators. The three classes were labeled according to acknowledgment and disclosure patterns for RC experiences: Class 1—*High unacknowledgment with ambivalence, High disclosure*; Class 2—*High acknowledgment, High disclosure*; and Class 3—*Hesitant acknowledgment, No disclosure*.

Figure 1 presents the conditional response probabilities for the 3-class model. The first class, *High unacknowledgment with ambivalence, High disclosure* is the largest, comprising 41% (*N* = 130) of the sample. It is characterized by very high likelihood of unacknowledgment (100%) with at the same time substantial recognition (82%) of certain RC experiences as violent. The likelihood of ambivalence about the experiences is moderate (35%), with high likelihood of disclosure to friends and family (86%) and moderate likelihood of disclosure to a professional (40%).

The second class, *High acknowledgment, High disclosure* represents 30% of the sample (*N* = 96). It is characterized by high probability of recognizing RC behaviors as violent (92%) and very low probability of unacknowledgment (0%) or ambivalence (0%). Similar to the first class, the probability of disclosure to family and friends is high (79%), with moderate probability of disclosure to a professional (36%).

The third class, *Hesitant acknowledgment, No disclosure*, is the smallest class, representing almost a third of the sample (29%; *N* = 91). It shows medium likelihood of unacknowledgment (56%), high likelihood of recognizing certain RC behaviors as violent (81%), and low likelihood of ambivalence (23%). This group stands out by the very low likelihood of disclosure to family and friends (0%) or professionals (0%).

### Research Question 2: LCA with Demographics and Psychosocial Factors

Multinomial logistic regressions were conducted to examine associations between acknowledgment and disclosure patterns and potentially related variables. The *High acknowledgment, High disclosure* class was selected as the reference class for comparison due to its distinct pattern. We then compared the two remaining classes to each other, using the *High unacknowledgment with ambivalence, High disclosure* class as the reference class as well. This procedure was repeated for the two remaining research questions. Table 3 presents the results.

**Table 3.** Associations Between Sociodemographic Variables, Social Identities, Social Support, Victimization Experiences and Health Consequences, and Patterns of Acknowledgment and Disclosure of RC (Multinomial Regressions).

Variables	*High Unacknowledgment with Ambivalence, High Disclosure* Class (Referent Class: *High Acknowledgment, High Disclosure* Class)		*Hesitant Acknowledgment, No Disclosure* Class (Referent Class: *High Acknowledgment, High Disclosure* Class)		*Hesitant Acknowledgment, No Disclosure* Class (Referent Class: *High Unacknowledgment with Ambivalence, High Disclosure*)	
	*OR*	95% CI	*OR*	95% CI	*OR*	95% CI
Demographics and psychosocial variables						
Age	0.962	[0.918, 1.007]	0.845	[0.940, 1.052]	1.034	[0.977, 1.095]
Sexual orientation						
Diversity (Ref.: Heterosexual)	1.191	[0.663, 2.139]	0.689	[0.289, 1.643]	0.579	[0.255, 1.311]
Education						
University (Ref.: High school and Cegep)	1.659	[0.869, 3.170]	1.142	[0.485, 2.690]	0.688	[0.303, 1.565]
Occupation						
Worker (Ref.: Student)	0.896	[0.475, 1.689]	2.123	[0.738, 6.109]	2.370	[0.874, 6.427]
Unemployed (Ref.: Student and Worker)	0.961	[0.179, 5.162]	0.530	[0.020, 13.768]	0.552	[0.025, 12.315]
Social identity						
Ethnocultural minority						
Yes (Ref.: No)	1.350	[0.393, 4.633]	1.541	[0.332, 7.159]	1.142	[0.293, 4.452]
Indigenous person						
Yes (Ref.: No)	0.689	[0.072, 6.573]	1.054	[0.072, 15.528]	1.531	[0.106, 22.165]
Presence of a disability						
Yes (Ref.: No)	0.818	[0.378, 1.768]	0.090***	[0.002, 4.923]	0.110***	[0.002, 5.591]
Social support						
Yes (Ref.: No)	0.752	[0.411, 1.375]	0.533*	[0.226, 1.258]	0.709	[0.315, 1.591]
Victimization experiences						
RC experiences						
Experience of contraceptive sabotage (Ref.: No)	0.540**	[0.290, 1.004]	0.792	[0.340, 1.848]	1.468	[0.648, 3.324]
Experience of pregnancy coercion (Ref.: No)	1.057	[0.496, 2.253]	0.865	[0.278, 2.688]	0.818	[0.282, 2.372]
Experience of control of pregnancy outcomes (Ref.: No)	0.384**	[0.145, 1.019]	0.054***	[0.000, 67.514]	0.142	[0.00, 164.484]
Number of RC experience	0.824	[0.664, 1.023]	0.744	[0.494, 1.122]	0.903	[0.610, 1.337]
Lifetime intimate partner violence						
Yes (Ref.: No)	0.525*	**[0.227, 1.212]**	0.338***	**[0.125, 0.915]**	0.643	[0.276, 1.498]
Health consequences						
Psychological consequences						
Yes (Ref.: No)	0.640	[0.229, 1.791]	0.233***	[0.079, 0.681]	0.364***	[0.140, 0.947]
Sexual health consequences						
Yes (Ref.: No)	1.016	[0.494, 1.858]	0.223***	[0.090, 0.477]	0.219***	[0.091, 0.460]
High level of PTSD symptoms						
Yes (Ref.: No)	0.915	[0.475, 1.764]	0.058***	[0.001, 2.788]	0.063***	[0.001, 2.886]

*Note*. CI=confidence interval; OR=odds ratio; RC=reproductive coercion.

*** *p* < .001; **p < .01; *p < .05.

Bold values denote statistical significance at the *p* < 0.01 or *p* < 0.05 level.

No differences in sociodemographic variables were found between classes, except for presence of a disability. Participants who reported a disability were less likely to be in the *Hesitant acknowledgment, No disclosure* class than the *High acknowledgment, High disclosure* class, *OR* = 0.090, *p* < .001. They were also less likely to be in the *Hesitant acknowledgment, No disclosure* class than the *High unacknowledgment with ambivalence, High disclosure* class, *OR* = 0.11, *p* < .001.

Social support was also a significant covariate. Participants with higher social support were two times less likely to be in the *Hesitant acknowledgment, No disclosure* class than the *High acknowledgment, High disclosure* class, *OR* = 0.533, *p* < .05.

### Research Question 3: LCA and Victimization Experiences

For lifetime RC, participants who experienced contraceptive sabotage were less prone to be in the *High unacknowledgment with ambivalence, High disclosure* class than the *High acknowledgment, High disclosure* class, *OR* = 0.540, *p* < .01. Participants who experienced control of pregnancy outcomes were less likely to be in the *High unacknowledgment with ambivalence, High disclosure* class, *OR* = 0.384, *p* < .01, or the *Hesitant acknowledgment, No disclosure* class, *OR* = 0.054, *p* < .001, than the *High acknowledgment, High disclosure* class. No differences were found between the three classes on the other RC type (pregnancy coercion) or the number of RC events experienced.

Lifetime prevalence of IPV was also associated with class membership. Participants who reported lifetime IPV experiences were almost two times less likely to be in the *High unacknowledgment with ambivalence, High disclosure* class than the *High acknowledgment, High disclosure* class, and almost three times less likely to be in the *Hesitant acknowledgment, No disclosure* class than the *High acknowledgment, High disclosure* class, respectively *OR* = 0.525, *p* < .05; *OR* = 0.338, *p* < .001.

### Research Question 4: LCA and Mental and Sexual Health

In terms of symptoms associated with RC experiences, participants with high levels of PTSD symptoms were less likely to be in the *Hesitant acknowledgment, No disclosure* class than the *High acknowledgment, High disclosure* class, *OR* = 0.058, *p* < .001. They were also less likely to be in the *Hesitant acknowledgment, No disclosure* class than the *High unacknowledgment with ambivalence, High disclosure* class, *OR* = 0.063, *p* < .001. For psychological and sexual health consequences, participants who reported more consequences were less likely to be in the *Hesitant acknowledgment, No disclosure* class than the *High acknowledgment, High disclosure* class, respectively *OR* = 0.233, *p* < .001; *OR* = 0.223, *p* < .001. Participants who reported more psychological and sexual consequences were also less likely to be in the *Hesitant acknowledgment, No disclosure* class than the *High unacknowledgment with ambivalence, High disclosure* class, respectively *OR* = 0.364, *p* < .001; *OR* = 0.219, *p* < .001.

## Discussion

This study aimed to determine patterns of acknowledgment of RC experiences and disclosure to family, friends, and healthcare professionals in a sample of 317 Canadians with the capacity to become pregnant. LCA results revealed that a three-class model provided the best data fit for the sample. We obtained a class labeled *High acknowledgment, High disclosure*, characterized by high levels of RC acknowledgment and informal disclosure. Two other classes were less clearly defined in terms of acknowledgment, although one of them showed high disclosure. The *High unacknowledgment with ambivalence, High disclosure* class was characterized by high levels of unacknowledgment and moderate ambivalence, while the third class, *Hesitant acknowledgment, No disclosure*, showed moderate levels of unacknowledgment, acknowledgment, and ambivalence. Both clearly stood out for their high levels of disclosure, which was higher for the *High unacknowledgment with ambivalence, High disclosure* class.

The *High unacknowledgment with ambivalence, High disclosure* class combined high ambivalence with high disclosure. This suggests that, due to ambivalence about identifying behaviors as violent and a tendency not to acknowledge them as such, these individuals chose to disclose their situation to family and friends to try to make sense of things (Bernstein & Newins, 2022; Orchowski et al., 2013). In fact, when individuals who have experienced sexual violence disclose what they have undergone, listeners’ reactions can influence how they perceive the experience. Thus, a negative and blaming attitude could make them feel guilty, and conversely, a positive and supportive response could help them shed feelings of blame and guilt (Harned, 2005; Lévesque & Rousseau, 2019; Littleton et al., 2006). In this sense, the reaction to RC disclosure could generate effects (i.e., blame or exoneration) akin to those generated by RC situations.

Similarly, participants in the *Hesitant acknowledgment, No disclosure* class may have had trouble identifying behaviors as problematic or violent, particularly in the absence of feedback from family and friends. Thus, being validated when they shared RC experiences would help them acknowledge the violence, whereas being refuted by others could prevent them from acknowledging behaviors as RC, and further, could contribute to lower their psychological well-being (Bujold & Lévesque, 2022; Orchowski et al., 2013).

Certain psychosocial characteristics appeared to moderate class membership. In our sample, participants who reported having a disability were 11 times more likely to be in the *High acknowledgment, High disclosure* class and nine times more likely to be in the *High unacknowledgment with ambivalence, High disclosure* class than the *Hesitant acknowledgment, No disclosure* class. Studies show that women who live with a disability present cumulative contexts of vulnerability to IPV (Sasseville et al., 2022). For instance, the lifetime prevalence of IPV was determined as two times higher for women with than without a handicap (Mitra & Mouradian, 2014; Statistics Canada, 2020). With respect to disclosure, these women would be at greater risk for negative feedback, and their family and friends would be more inclined to disbelieve them (Plummer & Findley, 2012), and especially when the women had intellectual disabilities (Rittmannsberger et al., 2022). Women with handicaps were also less likely to disclose violence to healthcare professionals, notably due to lack of access to adapted and volunteer services (Plummer & Findley, 2012). This concurs with the study by Alhusen et al. (2020), who held qualitative interviews with women with disabilities who had experienced RC. Their results showed that services for women with handicaps were neither adapted nor secure, which would prevent them from disclosing in order to obtain support. Our finding on this issue contradicts the literature, and further studies are needed to better understand the connections between victimization, acknowledgment of behaviors, and disclosure in women living with a handicap. Self-identification as a member of an ethnocultural minority does not moderate class membership, nor does not identifying as heterosexual.

Participants who reported higher social support were almost twice as likely to belong to the *High acknowledgment, High disclosure* class than the *Hesitant acknowledgment, No disclosure* class. It was demonstrated that disclosure generally led to supportive responses that positively impacted the victims’ well-being. This support helped them realize that certain behaviors were unacceptable (Harned, 2005; Lévesque & Rousseau, 2019).

The victimization experiences appeared to moderate class membership. Participants who reported contraceptive sabotage were more prone to belong to the *High acknowledgment, High disclosure* group than the two other groups. Here, we might suppose that it is easier to acknowledge behaviors that are more explicit and obvious. For example, nonconsensual condom removal and failure to withdraw before ejaculation are observable during and after sexual relations, which could make it easier to recognize the problematic nature of these behaviors. When a partner undermined contraception practices, some women relied on peer support to obtain access to other, less detectable methods (Moulton et al., 2021). Thus, witnessing RC behaviors and fearing for one’s sexual and reproductive health could spur some women to disclose what was happening so as to obtain help.

Similarly, the participants who were subjected to control of pregnancy outcomes were more likely to be in the *High acknowledgment, High disclosure* class. The behaviors related to control of pregnancy outcomes were mostly those recognized as IPV (e.g., threats and injuries). In such situations, some victims could have sought support as a coping strategy. When listeners consider these behaviors as violent, the victims can stop blaming themselves for what happened and gain a sense of empowerment (Fogarty et al., 2019).

Similar results were found on the lifetime prevalence of IPV. Participants who reported lifetime IPV experiences were about two times as likely to be in the *High acknowledgment, High disclosure* class than the *High unacknowledgment with ambivalence, High disclosure* class or the *Hesitant acknowledgment, No disclosure* class. The consequences of experiencing many situations of violence sometimes act as warning signs that help victims recognize the behaviors as violent. The severity and number of consequences can increase psychosocial and health needs, notably because the behaviors directly threaten sexual and reproductive health. Thus, when individuals realize the extent and persistence of the consequences of IPV on daily life, they may find it easier to acknowledge these behaviors as problematic (Cheng et al., 2022; Cho et al., 2020). It is therefore important to mention that the present study did not enable determining cooccurrence between lifetime RC experiences and lifetime IPV experiences.

The consequences for mental and sexual health appeared to moderate acknowledgment and disclosure. Participants with high levels of PTSD symptoms were about 16 times more likely to be in the *High acknowledgment, High disclosure* class than the two other classes. Regarding psychological consequences, participants who experienced more consequences were four times more likely to be in the *High acknowledgment, High disclosure* class than the *Hesitant acknowledgment, No disclosure* class. They were also two times more likely to be in the *High unacknowledgment with ambivalence, High disclosure* class than the *Hesitant acknowledgment, No disclosure* class. These results align with a study in which individuals who presented severe psychological distress were more likely to use support services (Cho et al., 2021). However, other studies reported the contrary: individuals who presented psychological (compared to physical) consequences of violence were less likely to seek support (Sabina et al., 2014; Wright & Johnson, 2009). This reluctance to seek help could be connected to the acknowledgment of the violent nature of the behaviors. One element that promotes acknowledgment of sexual violence and increases the risk of experiencing PTSD symptoms is the voicing of non-consent (Cook & Messman-Moore, 2018). Given that certain forms of RC are subtle and may be ongoing in a relationship, further studies are needed to explore relationships between acknowledgment and voicing non-consent in RC situations.

Participants who reported more sexual consequences were four times more likely to be in the High acknowledgment, High disclosure class or the High unacknowledgment with ambivalence, High disclosure class than the Hesitant acknowledgment, No disclosure class. A bidirectional association was identified between RC consequences and acknowledgment of violence. This suggests that more severe consequences could generate greater acknowledgment, which could lead in turn to greater consequences.

Although this study advances the understanding of the complex patterns of acknowledgment and disclosure of RC experiences, certain limitations must be considered. First, we should underscore that the sample is not representative of Québec’s population: the participants were relatively well educated and presented similar sociodemographic profiles in terms of ethnocultural membership. In addition, the patterns were determined based on a single time measure, which precludes identifying long-term changes in RC acknowledgment and disclosure. It also precludes making causal inferences between RC acknowledgment and disclosure. Acknowledgment of RC events could lead to higher disclosure, but high disclosure could also influence levels of acknowledgment. Further, longitudinal studies are recommended to determine the direction of this relationship.

### Implications and Recommendations

This study explores the relationships between RC experiences, acknowledgment of RC, and disclosure to friends, family, and professionals. It also highlights patterns of disclosure based on factors such as the type of RC experienced, the consequences, and the level of social support. These findings can guide the development of compassionate intervention strategies that encourage recognition of the harmful and violent nature of these behaviors, thereby promoting the well-being of victims-survivors.

Given that RC is not well understood by the general population, prevention initiatives are necessary to define the issue and promote recognition of such acts as violence. Awareness-raising campaigns targeted at various audiences, particularly victims and potential victims, are crucial. Many individuals are ambivalent about how to classify the behaviors they have experienced, and some do not recognize these behaviors as violent. Therefore, an information and awareness campaign that explains the different forms of RC, the criminal nature of certain behaviors, and where to seek support is needed.

The data also indicate that many victims turn to their immediate social circles to discuss their experiences. Hence, educating those around them should be a secondary focus. Additionally, awareness campaigns should target individuals who are likely to commit these acts.

However, acknowledgment alone is insufficient without resources to support victims afterward. Ongoing education for the general population and training for healthcare professionals to handle disclosures of violence is essential. This support can mitigate the consequences of these traumas. Continuing education for care staff, provided by professional associations, and enhanced training programs for health and social services professionals are recommended.

Despite varying levels of acknowledgment, many participants disclosed their experiences to family members, friends, or healthcare professionals. This study could not determine whether non-disclosure led to more severe or less identifiable consequences. Further research is needed to explore the relationships between the consequences, acknowledgment, and disclosure of RC situations.

## Supplemental Material

sj-docx-1-jiv-10.1177_08862605241259409 – Supplemental material for A Latent Class Analysis of Reproductive Coercion Experiences Based on Victim-Survivors’ Acknowledgment and Disclosure Patterns Supplemental material, sj-docx-1-jiv-10.1177_08862605241259409 for A Latent Class Analysis of Reproductive Coercion Experiences Based on Victim-Survivors’ Acknowledgment and Disclosure Patterns by Sylvie Lévesque, Arianne Jean-Thorn and Catherine Rousseau in Journal of Interpersonal Violence
