# Supplementary material for: A Latent Class Analysis of Reproductive Coercion Experiences Based on Victim-Survivors’ Acknowledgment and Disclosure Patterns
Source: J Interpers Violence. 2024 Jun 19;40(5-6):1360–86. doi: 10.1177/08862605241259409 (PMC11800699; doi:10.1177/08862605241259409)
Supplement: sj-docx-1-jiv-10.1177_08862605241259409 – Supplemental material for A Latent Class Analysis of Reproductive Coercion Experiences Based on Victim-Survivors’ Acknowledgment and Disclosure Patterns [file sj-docx-1-jiv-10.1177_08862605241259409.docx]

Supplementary material

1. Raw data

Participants responded to the questions on acknowledgment of reproductive coercion (RC) events only if they had reported one or more RC events. Participants were asked the following questions concerning acknowledgment:

In the previous questions, you reported having experienced, in your lifetime, certain events that took place in your intimate life.

Thinking about all the situations and behaviors that involved an intimate partner, in your opinion, were they:

|  | Please check all choices that apply. |
| --- | --- |
| **Q27a**  Miscommunications or misunderstandings? |  |
| **Q27b**  Lack of consideration for my choices/my person in the sexual relationship? |  |
| **Q27c**  Normal situation(s) in a couple or in an intimate relationship? |  |
| **Q27d**  About your partner’s desire to have a child? |  |
| **Q27e**  About your partner getting involved in birth control and family planning? |  |
| **Q27f**  Rape |  |
| **Q27g**  Domestic violence or control? |  |
| **Q27h**  Some type of crime, but not sure what type? |  |
| **Q27i**  I don’t know how to describe the event(s). |  |

Participants could check any or all of the responses that applied. Based on the literature on intimate partner violence, items a, c, d, and e were considered as unacknowledgment of violence; items b, f, g, and h as acknowledgment of violence; and item i as ambivalence.

1. Recoding and integration of Q27i with the other items.

Fictitious responses by 14 fictitious participants to 5 items (a, b, c, f, and i)

| **n** | **Q27a** | **Q27b** | **Q27c** | **Q27f** | **Q27i** |
| --- | --- | --- | --- | --- | --- |
| 1 | x |  | x |  |  |
| 2 | x |  |  |  |  |
| 3 | x | x | x | x |  |
| 4 |  | x |  |  | x |
| 5 |  |  |  |  |  |
| 6 |  |  | x |  | x |
| 7 |  |  |  |  | x |
| 8 |  |  |  | x | x |
| 9 | x | x |  |  |  |
| 10 | x |  |  |  | x |
| 11 |  | x |  |  |  |
| 12 |  |  | x |  |  |
| 13 |  |  |  |  |  |
| 14 | x |  |  |  | x |
| **TOTAL** | **6** | **4** | **4** | **2** | **6** |

In order to conduct analyses based on the same common denominator, we recoded the items while integrating the item (“I don’t know how to describe the event(s)”) with the other items.

In this case, we hypothesized that participants who checked Q27i meant only that they did not know whether the event(s) could be described by item Q27a or Q27b, Q27c or Q27d. For these participants, we attributed the “I don’t know” response (because they checked Q27i) for each item because they were unsure which ones to check. In addition, we hypothesized that participants who checked at least one of items Q27a, Q27b, Q27c, or Q27d combined with Q27i knew how to describe some of the events to some extent but were ambivalent about how to describe what had happened. We therefore considered that these participants were confused about which items to check “in addition” to the one they had already checked. Note that we hypothesized that participants who didn’t check any items had declined to respond. In this case, the only hypothesis would be a decline to respond, and in any case, the participants were not compelled to respond to any of the items.

The table below presents the recoded responses. As shown, there are no longer any responses to Q27i, and because it was integrated into the other items, we withdrew this item.

| **n** | **Q27a_r** | **Q27b_r** | **Q27c_r** | **Q27f_r** | **Q27i_r** |
| --- | --- | --- | --- | --- | --- |
| 1 | 1 | 0 | 1 | 0 |  |
| 2 | 1 | 0 | 0 | 0 |  |
| 3 | 1 | 1 | 1 | 1 |  |
| 4 | 998 | 1 | 998 | 998 |  |
| 5 | 999 | 999 | 999 | 999 |  |
| 6 | 998 | 998 | 1 | 998 |  |
| 7 | 998 | 998 | 998 | 998 |  |
| 8 | 998 | 998 | 998 | 1 |  |
| 9 | 1 | 1 | 0 | 0 |  |
| 10 | 1 | 998 | 998 | 998 |  |
| 11 | 0 | 1 | 0 | 0 |  |
| 12 | 0 | 0 | 1 | 0 |  |
| 13 | 999 | 999 | 999 | 999 |  |
| 14 | 1 | 998 | 998 | 998 |  |

0 = No

1 = Yes

998 = I don’t know

999 = Declined to respond

The table below presents the simple frequencies for each item after item Q27i and the declines to respond (999) were treated.

| Q27a_r | 1=Yes | 6 |
| --- | --- | --- |
|  | 0=No | 2 |
|  | 998=I don’t know | 4 |
|  | 999=Declined to respond | 2 |
|  | **n** | **14** |
|  |  |  |
| Q27b_r | 1=Yes | 4 |
|  | 0=No | 3 |
|  | 998=I don’t know | 5 |
|  | 999=Declined to respond | 2 |
|  | **n** | **14** |
|  |  |  |
| Q27c_r | 1=Yes | 4 |
|  | 0=No | 3 |
|  | 998=I don’t know | 5 |
|  | 999=Declined to respond | 2 |
|  | **n** | **14** |
|  |  |  |
| Q27f_r | 1=Yes | 2 |
|  | 0=No | 5 |
|  | 998=I don’t know | 5 |
|  | 999=Declined to respond | 2 |
|  | **n** | **14** |
|  |  |  |
| Q27i_r | **n** | **0** |

1. Creation of scores

Using the recoded items, we wanted to create scores for acknowledgment, unacknowledgment, and ambivalence. Note that a participant could be scored in all three classes at the same time. For the acknowledgment score, if participants responded Yes to at least one of the four items, an acknowledgment score of (1) was assigned, and (0) for a No response to all items. For unacknowledgment, a Yes response to at least one of the four items was scored as (1), and (0) for a No response to all items. For ambivalence, at least one I don’t know response to the eight items was scored as (1), and a score of (0) otherwise.

The table below presents a fictious example to illustrate the scoring.

| **n** | **Q27a_r**  **Unacknowledgment item** | **Q27b_r**  **Acknowledgment item** | **Q27c_r**  **Unacknowledgment item** | **Q27f_r**  **Acknowledgment item** | **Unacknowledgment score** | **Acknowledgment score** | **Ambivalence score** |
| --- | --- | --- | --- | --- | --- | --- | --- |
| 1 | 1 | 0 | 1 | 0 | 1 | 0 | 0 |
| 2 | 1 | 0 | 0 | 0 | 1 | 0 | 0 |
| 3 | 1 | 1 | 1 | 1 | 1 | 1 | 0 |
| 4 | 998 | 1 | 998 | 998 | 998 | 1 | 1 |
| 5 | 999 | 999 | 999 | 999 | . | . | . |
| 6 | 998 | 998 | 1 | 998 | 1 | 998 | 1 |
| 7 | 998 | 998 | 998 | 998 | 998 | 998 | 1 |
| 8 | 998 | 998 | 998 | 1 | 998 | 1 | 1 |
| 9 | 1 | 1 | 0 | 0 | 1 | 1 | 0 |
| 10 | 1 | 998 | 998 | 998 | 1 | 998 | 1 |
| 11 | 0 | 1 | 0 | 0 | 0 | 1 | 0 |
| 12 | 0 | 0 | 1 | 0 | 1 | 0 | 0 |
| 13 | 999 | 999 | 999 | 999 | . | . | . |
| 14 | 1 | 998 | 998 | 998 | 1 | 998 | 1 |

999 = Declined to respond; . = no score: declined to respond; 998 = I don’t know.
